# In Situ Preparation of rGO-Cement Using Thermal Reduction Method and Performance Study

**DOI:** 10.3390/ma17051209

**Published:** 2024-03-06

**Authors:** Jie Yao, Ao Guan, Wenqiang Ruan, Ying Ma

**Affiliations:** 1 School of Materials and Environmental Engineering, Shenzhen Polytechnic University, Shenzhen 518055, China; yaojie0125@163.com (J.Y.); 13304059224@163.com (A.G.); 2 School of Materials Science and Engineering, Shenyang Jianzhu University, Shenyang 110168, China; 3 Green Environmental Technology Research Institute, Shenzhen 518055, China; 4 School of Civil Engineering, Harbin Institute of Technology, Harbin 150090, China; ruanwenqiang8@163.com

**Keywords:** GO, rGO, cement-based materials, thermal reduction, freeze-drying

## Abstract

In this study, the combination of freeze-drying and high-temperature thermal reduction methods was employed to in situ prepare reduced graphene oxide (rGO)-Cement based on graphene oxide (GO)-Cement. The electrical conductivity and mechanical properties of the rGO-Cement were investigated. Microscopic analysis methods such as Raman spectra, X-ray photoelectron spectroscopy (XPS), and scanning electron microscopy (SEM) were used to confirm the successful transformation of GO-Cement to rGO-Cement. The research results demonstrated that with an increase in rGO content, the electrical resistivity of the rGO-Cement decreased first and then increased, reaching a percolation threshold at the dosage of 0.7 wt.%. The compressive strength and flexural strength of the rGO-Cement increased first and then decreased. The optimal dosage of rGO was 0.7%. The in situ preparation of rGO-Cement using the thermal reduction method holds a great potential for various applications, providing new ideas and methods for the modification and enhancement of cement materials.

## 1. Introduction

Graphene is a unique 2D nanomaterial widely used in various fields, possessing excellent mechanical, electrical, and thermal properties [1,2]. Graphene oxide (GO) and reduced graphene oxide (rGO) are the primary derivatives of graphene [3]. In recent years, GO and rGO have been extensively utilized in cement-based materials to enhance their mechanical properties, electrical conductivity, and durability [4,5,6]. GO, due to its abundance of oxygen-containing functional groups on the GO sheet, exhibits excellent dispersibility in aqueous solutions [7]. This characteristic enables GO to interact well with cementitious materials, resulting in the enhanced properties of GO-modified cementitious composites [8]. However, the presence of oxygen-containing functional groups introduces numerous defects, leading to a loss of conductivity in GO [9]. To overcome this issue and improve the conductivity of composites, many researchers have used various methods to convert GO into rGO in cement-based composites [10]. This transformation is crucial for the widespread application of graphene in cement-based composites.

Currently, there are mainly two methods for converting GO to rGO: chemical reduction and thermal reduction. GO was prone to substantial deoxygenation in an alkaline environment (NaOH/KOH) [11]. It was found that cement-based composites can provide an alkaline environment for GO during the hydration process, enabling the conversion of C=O functional groups to C-O bonds and eliminating residual oxygen-containing functional groups [12,13]. The chemical reactions between GO and hydration products (CSH, Ca(OH)_2_, and AFt) in the cement paste can even produce more CaCO_3_ through the release of CO, CO_2_, and Ca(OH)_2_ [14]. These interactions not only promote cement hydration but also densify the microstructure, thereby enhancing the mechanical properties. The reduction temperature and duration have been reported as crucial factors influencing the extent of GO reduction [15,16]. Under alkaline conditions, the degree of reduction increases with temperature and time [16,17,18]. Common reducing agents include N_2_H_4_, H_2_S, NaBH_4_, and B_2_H_6_ [19]. However, the in situ reduction of GO in cement-based composites is somewhat limited due to the toxicity of these reducing agents [20,21,22].

On the other hand, high-temperature thermal reduction is suitable for the in situ preparation of rGO-Cement composites by deoxygenating GO at a high temperature [23,24,25]. It has been reported that GO could be well dispersed in cement particles for high temperature thermal reduction and the in situ preparation of rGO-Cement. The incorporation of rGO-Cement in cementitious composites can increase the compressive strength and durability [15]. The main challenge of employing the thermal reduction method for the in situ preparation of rGO-Cement lies in the dispersion of GO in cement particles. Using water as the solvent of GO suspension for dispersion in cement particles will inevitably trigger cement hydration. Thus, Ruttanapun et al. [26] used an organic solvent (acetone) to prepare GO suspension for dispersion in calcium aluminate (GO@CA) cement, which was then converted to rGO@CA cement powder at 300 °C. They observed that the collaborative impact of electron transfer and diffusion at the interface between cement particles and rGO augmented the conductivity of cementitious composites incorporating rGO-cement. In addition, Zhong et al. [27] compared the methods of traditional/microwave drying, vacuum drying, and freeze-drying for inhibiting cement hydration and found that freeze-drying is an effective method for preventing cement hydration. Currently, there are fewer and more complex methods for adsorbing carbon materials onto the surface of cement particles [28], such as chemical vapor deposition (CVD) [29]. In summary, in the silicate cement system, GO exhibits a better dispersibility than graphene/rGO [30]. Therefore, graphene oxide in the form of water solution can be more uniformly adsorbed onto the surface of cement particles. It is well known that cement is a water-hardening binder material [31], initiating a rapid reaction when in contact with water. Freeze-drying technology can quickly inhibit the hydration of cement [32]. The electrical conductivity of rGO is superior to that of GO. By using a high-temperature thermal reduction method, GO can be reduced to rGO, imparting a better electrical conductivity to cement.

In this study, we propose a novel method for the in situ preparation of rGO-cement based on freeze-drying and high-temperature thermal reduction. The ultrasonic dispersion method is employed to mix the GO suspension and cement particles at a high speed (1000 r/min). The GO-Cement mixture is subjected to freeze-drying, followed by the high-temperature thermal reduction, for the in situ preparation of rGO-Cement. The cement particles before and after reduction are characterized to verify the effectiveness of the reduction method. The electrical conductivity and mechanical properties of cementitious composites incorporated in rGO-Cement are further tested.

## 2. Raw Materials and Experimental Methods

### 2.1. Raw Material

**Figure 1:**
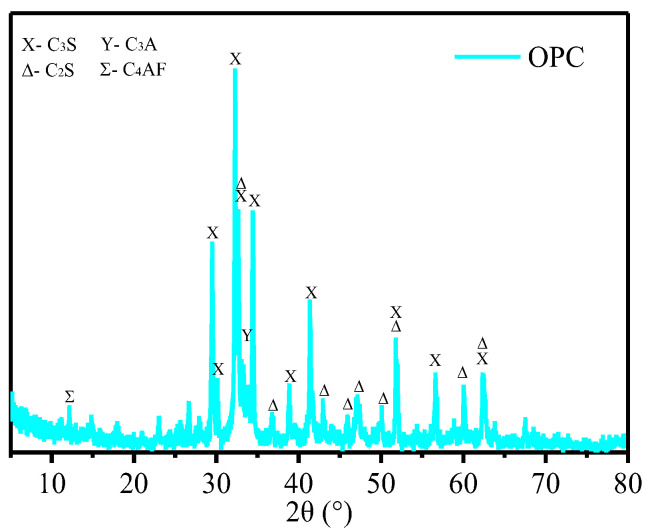
XRD pattern of cement.

The cement used was a type of P O 42.5 R Portland cement produced by Anhui Hailuo Cement Company Limited (Wuhu, China). The chemical composition and crystalline mineral phases of OPC are summarized in Table 1 and Figure 1, respectively. The GO aqueous suspension was provided by Angxing New Carbon Materials Co., Ltd. (Changzhou, China). The original GO solution has a concentration of 7 mg/mL, with a thickness of 1 nm for a single layer of GO and a lateral size ranging from 5 to 40 μm. A high-performance polycarboxylate superplasticizer was used as a surfactant to enhance the dispersibility of GO in cement slurry [13]. The solid content of the water-reducing admixture was 28%.

### 2.2. Sample Preparation

**Figure 2:**
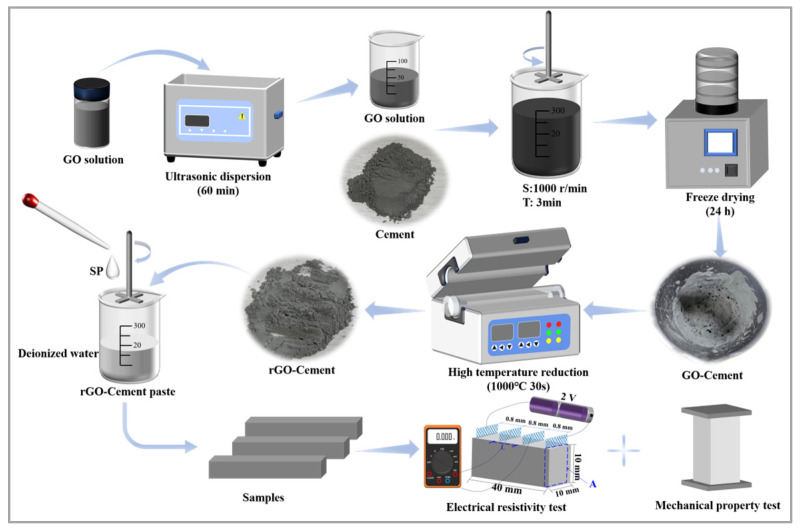
Preparation of rGO-Cement powder and its pure slurry.

The procedure of preparing (GO/rGO)-Cement and the cement paste with (GO/rGO)-Cement are shown in Figure 2. GO suspension with various concentrations were prepared by mixing the original concentrated GO solution with deionized water. The diluted GO suspension was ultrasonically dispersed for 60 min to achieve a good dispersion. The well-dispersed GO suspension was then mixed with cement at a high speed of 1000 rpm for 3 min. The mixture was quickly transferred to a freeze-dryer containing liquid nitrogen and was freeze-dried for 24 h, which produced the GO-Cement particles. Subsequently, the GO-Cement particles were rapidly heated up to and kept under 1000 °C for 30 s in a muffle furnace for the conversion to rGO-Cement particles. The produced rGO-Cement particles were then characterized. The GO-Cement and rGO-Cement paste specimens were prepared using the produced GO-Cement and rGO-Cement powder, and their electrical resistivity and mechanical properties were studied. The water/binder ratio of the cement paste was 0.35, and the sample was cured in the standard curing room (T = 20 °C ± 2, RH = 95%) to the specified age.

### 2.3. Performance Testing

#### 2.3.1. X-ray Fluorescence (XRF) Characterization

The chemical element composition and the content of ordinary Portland cement were determined using the Thermo Scientific ARL Perform’X X-ray Fluorescence instrument (Thermo Fisher Scientific, Waltham, MA, USA). A specific quantity of the cement sample was passed through a 200-mesh square sieve and then dried in a vacuum-drying oven at 60 °C for 24 h. The sample was tested after it underwent compression molding.

#### 2.3.2. Raman Spectra Analysis

The experiment employed the Horiba LabRAM HR Evolution Raman spectrometer (Horiba, Kyoto, Japan), produced in Japan, for qualitative analysis of the samples. The laser power used for testing was 5%, with a laser wavelength of 532 nm, and a measurement time of 10 s. The changes in peak intensity of the Raman spectrum allowed for the analysis of functional groups before and after sample reduction, while the changes in peak width enabled the analysis of material disorder and defect changes in the samples.

#### 2.3.3. X-ray Photoelectron Spectroscopy (XPS) Characterization

The experiment used the Thermo Scientific K-Alpha XPS (Thermo Fisher Scientific, Waltham, MA, USA), from the United States, to perform XPS analysis on the samples. It was used to identify the material composition of GO-Cement before and after reduction. The intensity changes of the C=O, C-C, O-C=O, and C-O peaks in the cement powder before and after reduction were analyzed to investigate whether the GO-Cement sample was successfully reduced to rGO-Cement.

#### 2.3.4. X-ray Diffraction (XRD) Characterization

The Bruker D8 XRD instrument was employed to analyze the hydration products of GO-Cement paste and rGO-Cement paste after 28 days of curing. The sample is immersed in a plastic tube containing anhydrous ethanol to halt the cement’s hydration process. After a 3-day soaking period, the sample is extracted and dried in a vacuum-drying oven at 45 °C for 24 h. The bulk samples are subsequently ground into a powder using an agate mortar and screened through a 200-mesh screen for the XRD test. This test used Cu Kα radiation in the 2θ region between 5° and 70° with a scanning rate of 10°/min. The XRD images were analyzed utilizing the Jade 6 software. The utilized PDF cards are as follows: C_3_S: PDF# 86-0402, C_2_S: PDF# 83-0460, C_3_A: PDF# 38-1429, C_4_AF: PDF# 71-0667, Ca(OH)_2_: PDF# 50-0008, and AFt: PDF#41-1451.

#### 2.3.5. Scanning Electron Microscope (SEM) Characterization

The microstructures of GO and rGO exhibit significant differences, and the surface of cement powder with GO/rGO can be easily distinguished through scanning electron microscopy (SEM). The Gemini SEM 300 field SEM microscope (Carl Zelss Co., Ltd., Jena, Germany) was employed to study the microstructure of GO-Cement and rGO-Cement particles. First, GO-Cement powder and rGO-Cement powder were placed in a vacuum-drying oven at 45 °C for 24 h. Subsequently, the powder was uniformly spread on conductive adhesive and spray platinum on the sample surface for clearer observation. The acceleration voltage used during testing was 15 kV, with a working distance of 10 mm.

### 2.4. Electrical Resistivity Testing

**Figure 3:**
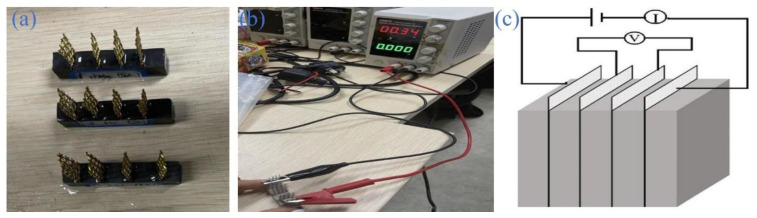
(**a**) Schematic diagram of resistivity test sample, (**b**) schematic diagram of voltage regulator power supply, and (**c**) schematic diagram of resistivity test principle.

As illustrated in Figure 3, the electrical resistivity of mortar was measured using a four-electrode method. The sample used for resistivity testing is shown in Figure 3a. The Dc-regulated power supply was used as the power source (2 V), and a multimeter was used to measure the voltage (U) and current (I) across the middle two electrodes. The current and voltage readings were taken after a uniform waiting period of 60 s. The resistivity (ρ, Ω cm) of the mortars can be calculated using Equation (1):

(1) $$\rho =\frac{U}{I}\times \frac{A}{L}$$

where U denotes voltage (V), I represents current flowing through the mortars (A), A denotes the cross-sectional area of the mortar (cm^2^), and L represents the distance between the two inner electrodes (cm).

### 2.5. Mechanical Property

The compressive and flexure strength tests of the cement paste specimens were carried out in accordance with the Chinese standard GB/T 17617-2007 [33]. The sample size for the flexural and compressive strength was 10 cm × 10 cm × 40 cm. The experimental testing instrument was SFL-50KNAG, a constant-loading cement compressive and flexure integrated testing machine manufactured by SHIMADZU Experimental Equipment Co., Ltd., Tokyo, Japan. The specimens were cured in a standard curing room at a temperature of 20 °C ± 2 and a relative humidity of 95% for 3 days, 7 days, and 28 days. The compressive strength of rGO-cement paste was measured on a compression test machine with the loading rate of 1200 N/s. Three samples were tested for each group, and their average strength values were calculated, along with the standard deviation.

## 3. Results and Discussion

### 3.1. Molecular Structure of GO-Cement and rGO-Cement

#### 3.1.1. Raman Spectra Analysis

**Figure 4:**
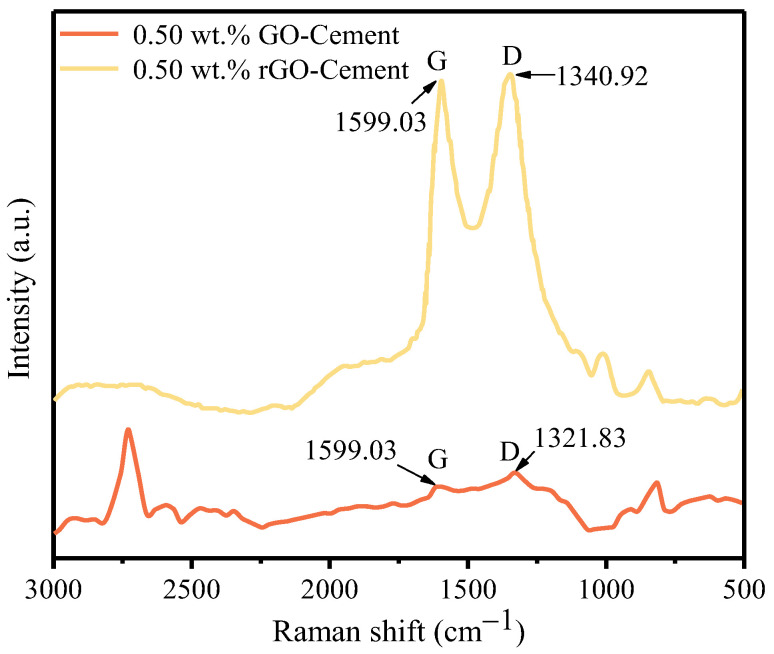
Raman spectra of GO-Cement particles and rGO-Cement particles at 0.50 wt.% GO.

Figure 4 shows the Raman spectra of GO-Cement powder and rGO-Cement powder with a 0.50 wt.% GO content. The D peak and G peak, situated at approximately 1300 cm^−1^ and 1580 cm^−1^, respectively, serve as distinctive Raman peaks for carbon atom crystals. The D peak signifies lattice defects in carbon atoms, while the G peak corresponds to the in-plane stretching vibration of SP^2^-hybridized carbon atoms [34]. In the GO/rGO Raman spectrum, two characteristic peaks appeared at 1350–1650 cm^−1^, representing the D peak and G peak of the GO/rGO structure [35].

As shown in Figure 4, in the 0.50 wt.% GO-Cement sample, characteristic peaks of GO, namely, the D peak and G peak [36], were observed at 1322 cm^−1^ and 1600 cm^−1^ [37], indicating that the cement powder adsorbed a certain amount of GO after freeze-drying. After being annealed at 1000 °C for 30 s, distinct characteristic peaks appeared at 1341 cm^−1^ and 1600 cm^−1^, indicating that high-temperature thermal reduction could transform GO into rGO [38]. In the study by Hu et al. [39], it was observed that GO could gradually decompose at 800 °C, reducing the oxygen functional groups on the surface and resulting in the formation of rGO. Therefore, it could be concluded that in this study, rGO-Cement powder was synthesized in situ through a high-temperature thermal reduction process. It was worth noting that compared to GO-Cement sample, the G peak of the rGO-Cement sample did not show a significant shift, while the D peak shifted to higher wavenumbers. This indicates that the intensity ratio of the D and G bands (ID/IG) ratio [40] of rGO-Cement powder is higher than that of GO-Cement powder, as the decomposition of oxygen functional groups on the surface of GO at high temperatures released gases and increased the vibrational energy of the system, leading to a decrease in crystallinity and an increase in defects. In the study by Sharma et al. [38], similar results were also observed. It could also be inferred that the GO-Cement particles successfully converted to rGO-Cement particles through the high-temperature reduction.

#### 3.1.2. XPS Analysis

**Figure 5:**
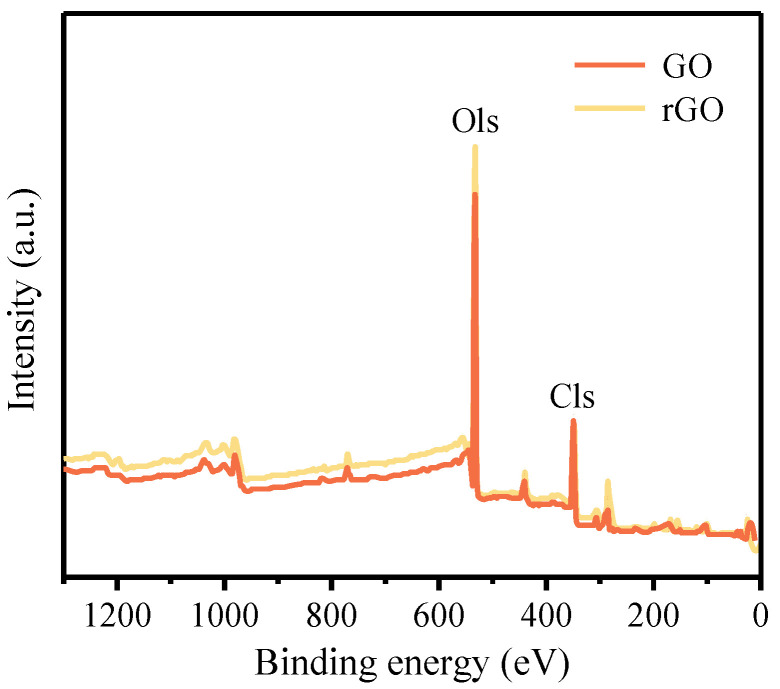
XPS spectra of 0.50 wt.% GO-Cement and 0.50 wt.% rGO-Cement.

XPS was utilized for the further analysis of changes in oxygen content and oxygen functional groups in the GO-Cement powder before and after the high-temperature reduction. Figure 5 presents the full spectra of GO-Cement powder before and after reduction. As shown in Figure 5, for the 0.50 wt.% GO-Cement, the Cls (At.%) and Ols (At.%) values were 15.72% and 84.28%, respectively, with an O/C ratio of 5.361. For the 0.50 wt.% rGO-Cement, the C1s (At.%) and O1s (At.%) values were 19.85% and 80.15%, respectively, with an O/C ratio of 4.038. It could be observed that a significant deoxygenation process occurred after the high-temperature reduction [41], which is consistent with the results of the Raman spectroscopy analysis. The conductivity of graphene was closely related to the number of C=C bonds, and the C/O ratio could be used to measure the conductivity of GO [42]. The C/O ratio of rGO was 0.248, which was higher than that of GO (0.187), indicating that the rGO-Cement paste would potentially possess a higher conductivity than the GO-Cement paste.

**Figure 6:**
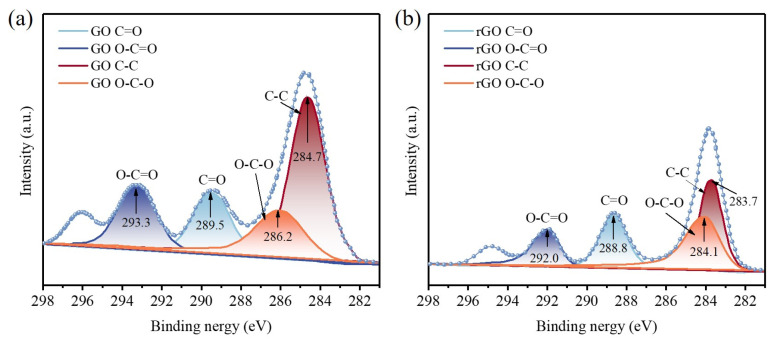
C1s spectra of (**a**) 0.50 wt.% GO-Cement and (**b**) 0.50 wt.% rGO-Cement.

Figure 6 displays the fitted Cls peaks of GO-Cement before and after the thermal reduction. It could be observed that the intensity of the C-C bond increased in the 0.50 wt.% rGO-Cement, while the peaks of C=O, O-C=O, and O-C-O weakened, indicating the decomposition of most oxygen functional groups [43]. Due to the significantly large surface area of GO, it readily adsorbed onto the surface of cement particles, affecting the complete decomposition of functional groups during the thermal reduction process. The reduction in intensity was more pronounced for the C=O and O-C=O peaks, indicating easier thermal decomposition [44]. Based on the results of Raman spectra and XPS, it can be concluded that the freeze-drying method is capable of addressing the dispersion issue of GO in cement particles and preparing GO-Cement, and the high-temperature thermal reduction (1000 °C) method enabled the conversion of the GO-Cement to the rGO-Cement.

#### 3.1.3. Microstructural Morphology of (GO/rGO)-Cement

**Figure 7:**
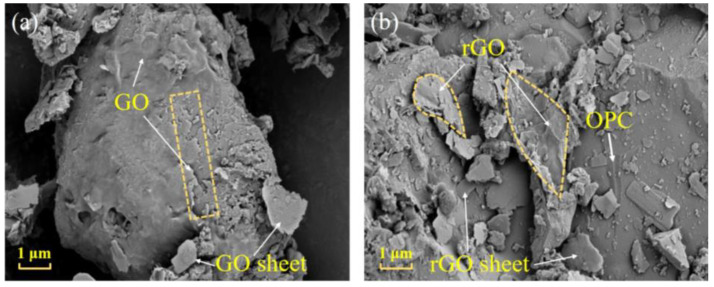
SEM images of (**a**) 0.50 wt.% GO-Cement and (**b**) 0.50 wt.% rGO-Cement.

The binding form of GO/rGO with cement particles had a significant impact on the modified cement. The microstructure of GO-Cement and rGO-Cement samples were investigated with SEM. As shown in Figure 7a, it could be observed that through the freeze-drying treatment, GO tightly adhered to the surface of large cement particles, forming “core-shell” structures and creating a dense encapsulation layer. However, it was also noticed that a part of the GO was unevenly dispersed on the cement surface, possibly due to the friction between particles during the preparation process, resulting in a rough texture. Adsorption in the form of GO sheets onto the surface of cement particles was also noted. The amount of GO encapsulated on the surface of extremely small particles was relatively low, indicating that at a 0.50 wt.% dosage, it was insufficient to fully encapsulate all cement particles. In Figure 7b, the surface of the dense encapsulation layer exhibits wrinkles, suggesting that the decomposition of oxygen functional groups results in the bending and deformation of the edges of rGO structures. Surface folds, a characteristic of graphene, are evident, i.e., the shape outlined in the yellow box on the right in Figure 7b. Additionally, it is observed that rGO is adsorbed as small flakes on the surface of cement particles, as indicated by the shape marked in the yellow box on the left in Figure 7b. This elucidates the reduction in the quantity of C-C bonds following high-temperature thermal reduction. This reflects the thermal decomposition process in which large sheet-like GO structures decompose into smaller flake-like structures, accompanied by a decrease in crystallinity. The presence of rGO in the microstructure further confirms the successful reduction of GO to rGO.

Through the high-temperature reduction process, GO on the surface of cement particles could be converted to rGO, forming a tightly encapsulating layer. The high-temperature reduction process led to the decomposition of oxygen functional groups and structural deformations, which helped enhance the conductivity of rGO-Cement [45]. Additionally, the microstructure analysis confirmed the presence of rGO and revealed the binding forms between GO/rGO and cement particles.

During the high-temperature thermal reduction process, the Raman spectrum of GO molecules exhibits the appearance of D and G peaks, as well as the decomposition of oxygen functional groups in the molecular structure (C=O, O-C=O). Simultaneously, there is a change in the microscopic structure, providing a molecular-level confirmation of GO being reduced to rGO. Additionally, through the changes in microscopic morphology, the similarity to graphene is revealed. This series of observations confirms the feasibility of using the high-temperature thermal reduction method for the in situ preparation of conductive cement.

### 3.2. Electrical Conductivity of the rGO-Cement Paste

**Figure 8:**
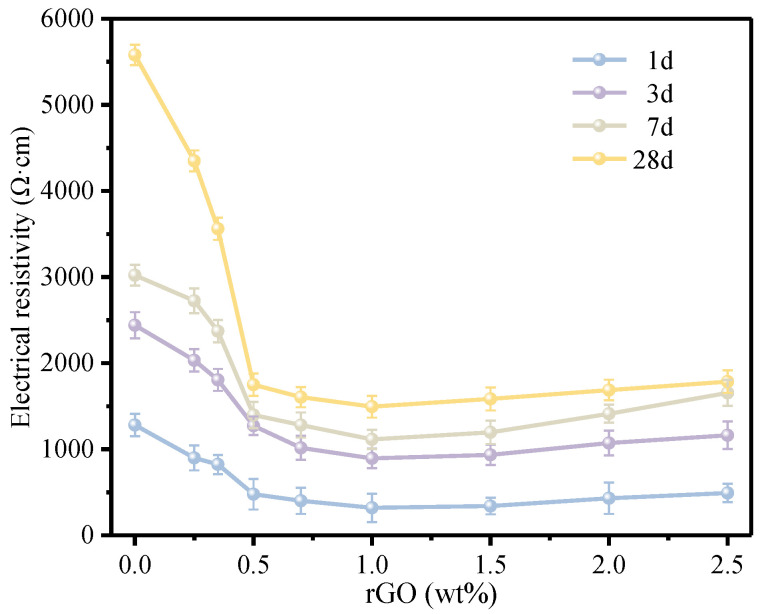
Effects of GO-doping amounts on the resistivity of rGO-Cement matrix composite.

rGO could impart excellent conductivity to cement-based materials, and its contribution to the conductivity of cement could be reflected through conductivity tests, which could also determine the permeation threshold of rGO-Cement. In the rGO-Cement paste, the initial content of GO in cement powder was 0, 0.25 wt.%, 0.35 wt.%, 0.50 wt.%, 0.70 wt.%, 1.00 wt.%, 1.50 wt.%, 2.00 wt.%, and 2.50 wt.%, respectively. After the high-temperature reduction, rGO-Cement pastes with different contents were obtained. Figure 8 presents the resistivity of rGO-Cement pastes with different contents by curing for 1, 3, 7, and 28 d.

As shown in Figure 8, the addition of rGO significantly decreased the electrical resistivity of the cement paste with its dosage. The incorporation of rGO enhanced the conductivity of the cement paste, although the resistivity of the rGO-Cement samples did not exhibit a monotonic relationship with the rGO content. In the first stage, at lower rGO contents (0.25–0.50 wt.%), the resistivity decreased gradually after curing for 1 and 3 d. For the rGO content of 0.50 wt.%, the resistivity was reduced by 61.7% compared with pure cement paste after curing for 1 d, and 48.0% compared with pure cement paste after curing for 3 d. Cement-based composites primarily conducted electricity through ionic conduction, hole (contact and tunneling effects), and electronic conduction. At this stage, the rGO content gradually increases, and the electrical conductivity of the cement slurry is significantly improved. But the formed conductive network is not stable enough. In the second stage (GO content of 0.70–1.00 wt.%), the trend of the resistivity reduction became more pronounced. At the rGO content of 1.00 wt.%, the percolation threshold was reached, and the electrical conductivity was optimal. In the study by Zhai et al. [46], when the doping concentration of rGO exceeded 1.00%, the decrease in resistivity slowed down with the increasing concentration, indicating that it reached percolation threshold. Compared to the pure cement paste, the resistivity reduction after curing for 1, 3, 7, and 28 d was 75.4%, 63.3%, 63.1%, and 73.1%, respectively. This was because rGO gradually formed a complete conductive network in the cement paste matrix, with contact conduction becoming the primary mode of conduction [47]. With the high charge carrier mobility of rGO, the resistivity of the cement paste was significantly reduced, leading to a remarkable improvement in the conductivity of the cement material [48]. In the third stage (GO content of 1.50–2.50 wt.%), the resistivity slightly increased compared to the 1.00 wt.% GO content. The resistivity trend for specimens cured for 7 and 28 d was the same as that of 1 and 3 d, showing a decreasing trend followed by a slight increase with an increase in the rGO content. Therefore, it could be inferred that the percolation threshold of the rGO-Cement paste in this system was 1.00 wt.%. Due to the high conductivity of rGO and the dispersion achieved through freeze-drying and high-temperature reduction, rGO was uniformly dispersed in the cement-based material. This led to the formation of a spatial conductive network at a rGO content of 1.00 wt.%, providing the rGO-Cement based composite material with a low content and high-conductivity characteristics.

### 3.3. Mechanical Properties of rGO-Cement Paste

**Figure 9:**
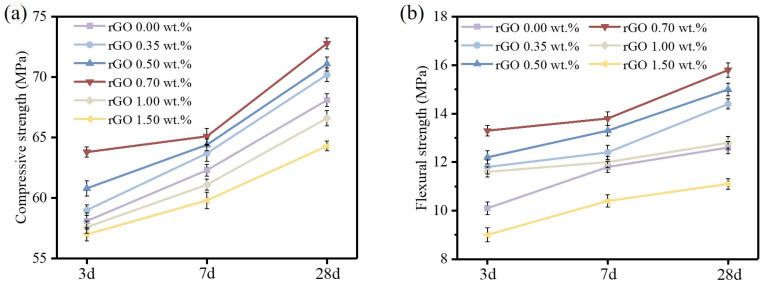
Mechanical properties of cement with different amounts of rGO: (**a**) the compressive strength and (**b**) the flexural strength.

As a nanofiller materials, rGO in cement-based paste exhibited excellent performances in promoting cement hydration and improving the microstructure. As shown in Figure 9a, the compressive strength of rGO-Cement paste increased with the curing age. With the increase in the amount of rGO doping, compressive strength initially rises and then declines, following a pattern similar to the findings of Zhang et al. [49]. It is also noted that the decrease in compressive strength is attributed to the aggregation of rGO.The addition of a small amount of rGO (0.35–0.70 wt.%) consistently resulted in a higher compressive strength compared to the pure cement samples, especially for rGO-Cement paste with a GO content of 0.70 wt.%, which exhibited the highest compressive strength. At a curing age of 3 d, the compressive strength of the rGO-Cement samples with GO contents of 0.35 wt.%, 0.50 wt.%, and 0.70 wt.% was increased by 1.5%, 4.6%, and 9.8%, respectively, compared to the pure cement. At a curing age of 28 d, the compressive strength of the rGO/Cement samples with GO contents of 0.35 wt.%, 0.50 wt.%, and 0.70 wt.% was increased by 3.1%, 4.4%, and 6.9%, respectively, compared to the pure cement. However, when the GO content (0.70–1.50 wt.%) increased, the compressive strength of the rGO-Cement samples was consistently lower than that of the pure cement samples. With a GO content of 1.50 wt.%, the compressive strength of the rGO-Cement samples decreased by 1.9%, 4.0%, and 5.6% at 3, 7, and 28 d, respectively, compared to the pure cement samples. From the experimental results, it could be concluded that low contents of GO content (0.35–0.70 wt.%) had a relatively better effect on improving the early compressive strength of rGO-Cement.

As shown in Figure 9b, the flexural strength of rGO-Cement continuously increased with the age of curing. The maximum flexural strength was achieved at a rGO content of 1.00 wt.%, but when the rGO content was further increased up to 1.50 wt.%, the flexural strength decreased compared to the pure cement specimens. This indicated that the excessive rGO led to agglomeration, making it extremely difficult to achieve a uniform dispersion of graphene throughout the entire cement matrix, resulting in a decrease in mechanical properties. At a curing age of 3 d, the flexural strength of rGO-Cement with 0.35 wt.%, 0.50 wt.%, 0.70 wt.%, and 1.00 wt.% rGO was increased by 3.0%, 5.0%, 7.0%, and 13.8%, respectively, while the flexural strength of the specimen with 1.50 wt.% GO was decreased by 5.0%. At the curing age of 28 d, the flexural strength of rGO-Cement with 0.35 wt.%, 0.50 wt.%, 0.70 wt.%, and 1.00 wt.% rGO was increased by 1.6%, 2.4%, 4.8%, and 9.5%, respectively, while the flexural strength of the specimen with 1.50 wt.% GO was decreased by 3.2%. Similarly, the addition of a small amount (0.35–1.00 wt.%) of rGO had a better effect on improving the early flexural performance of rGO-Cement.

### 3.4. XRD Phase Analysis of (GO/rGO)-Cement Paste

**Figure 10:**
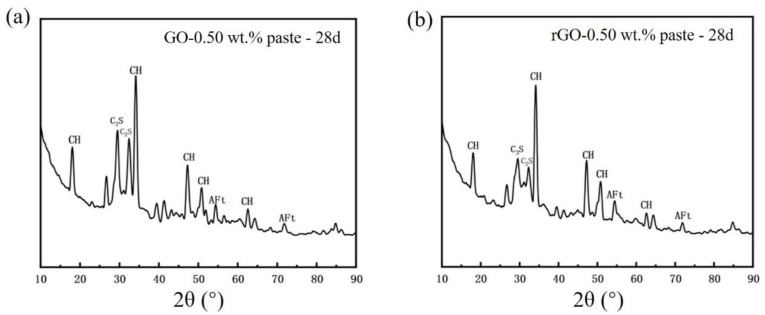
XRD patterns of cement at 28d: (**a**) 0.50 wt.% GO-Cement and (**b**) 0.50 wt.% rGO-Cement.

Due to the promoting effect of rGO on the cement hydration, XRD comparative analysis was conducted to examine the phase compositions of cement and rGO-Cement at 28 d. Figure 10 shows the XRD patterns of pure cement and rGO-Cement paste with a 0.5 wt.% rGO content. At 28 d, the major hydration products observed were calcium hydroxide (CH), ettringite (AFt), tricalcium silicate (C_3_S), and dicalcium silicate (C_2_S) [13]. It could be observed that the peaks corresponding to C_3_S and C_2_S were significantly reduced when rGO was added, indicating a higher degree of cement hydration. CH, as an early hydration product in the cement system, gradually decomposed during the later stages of hydration, and the CH peak in the rGO-Cement paste system was slightly lower. This suggested that rGO promoted the rate of cement hydration to some extent [15], reflecting an increase in the compressive and flexural strength with the increasing rGO content. The in situ preparation of rGO on cement particles and its encapsulation on the surface of cement particles contribute to better cement hydration. Additionally, the rapid freeze-drying process did not exhibit any negative effects resulting from the free water present in the GO solution. Therefore, the combination of freeze-drying and the high-temperature reduction to produce rGO-Cement paste proved to be an effective method for cement modification.

## 4. Conclusions

After the rapid mixing of GO solution and cement powder, the GO-Cement mixture maintained a non-hydrated state during the freeze-drying process without affecting the normal hydration of cement. GO-Cement was prepared in this manner, and subsequently, the high-temperature thermal reduction facilitated the significant reduction of GO-Cement to rGO-Cement. Therefore, the transformation process from GO-Cement to rGO-Cement during the thermal reduction process was studied using Raman spectroscopy, XPS, and SEM analysis methods. The electrical conductivity and mechanical properties of the cement materials were investigated with different rGO contents. The main conclusions drawn from the study are as follows:

(1) After high-temperature thermal reduction, the Raman spectra exhibit the distinctive D and G characteristic peaks of graphene. The bonding energy of the O-C=O bond, O-C-O bond, and C=O bond weakens, and their quantity decreases, leading to the high-temperature decomposition of numerous oxygen-containing functional groups. This proves the conversion of GO on the cement surface to rGO. The reduction in the number of C-C bonds indicates the breakdown of large sheets of GO structure into smaller sheets of rGO. The microscopic morphology on the cement surface transitions from a dense coating layer to fragmented small sheets, and the folding is also a characteristic feature of graphene. The freeze-drying and high-temperature thermal reduction method for the in situ preparation of rGO-Cement offers the advantages of simplicity and efficiency, facilitating a better dispersion of rGO within cementitious materials.
(2) The rGO coated on the surface of cement particles significantly enhances the electrical conductivity of rGO-Cement paste, reducing its electrical resistivity from 5.58 × 10^3^ Ω·cm to 1.49 × 10^3^ Ω·cm, a decrease of 73.3%. When 0.70 wt.% of rGO is coated on the surface of cement particles, the penetration threshold can be achieved. The best electrical conductivity is observed at a rGO content of 1.0 wt.%.
(3) The presence of rGO on the surface of cement particles promotes the hydration of cement, contributing to the enhancement of compressive and flexural strength. However, an excessive coating of rGO around cement particles hinders the diffusion of ions, impeding the formation of hydration products and consequently reducing the mechanical properties. Therefore, with an increase in the rGO content, the mechanical performance initially increases and then decreases. The optimal mechanical properties were obtained when the content of rGO was 0.70 wt.%. At a hydration age of 28 days, the contents of C_3_S and C_2_S in the phase of rGO-Cement are lower than those in GO-Cement. The later-stage hydration of rGO-Cement is more thorough, leading to an improvement in the mechanical properties of the paste.

## Figures and Tables

**Table 1:** Chemical compositions of cement (mass %).

CaO	SiO_2_	Al_2_O_3_	SO_3_	MgO	Fe_2_O_3_	K_2_O	TiO_2_	LOI
49.7%	24.1%	9.5%	5.7%	3.4%	2.7%	0.9%	0.3%	3.7%

## Data Availability

Data are contained within the article.

## References

[1] Geim A.K., Novoselov K.S. (2007). The rise of graphene. Nat. Mater..

[2] Mohammed A., Sanjayan J.G., Duan W.H., Nazari A. (2015). Incorporating graphene oxide in cement composites: A study of transport properties. Constr. Build. Mater..

[3] Ho V.D., Gholampour A., Losic D., Ozbakkaloglu T. (2021). Enhancing the performance and environmental impact of alkali-activated binder-based composites containing graphene oxide and industrial by-products. Constr. Build. Mater..

[4] Polverino S., Del Rio Castillo A.E., Brencich A., Marasco L., Bonaccorso F., Morbiducci R. (2022). Few-Layers Graphene-Based Cement Mortars: Production Process and Mechanical Properties. Sustainability.

[5] Janowska-Renkas E., Zdrojek M., Kozioł M., Kaliciak-Kownacka A. (2023). Effect of composition of geopolymer composites containing fly ash and waste glass powder on their durability and resistivity demonstrated in presence of a nanocarbon additive in a form of graphene. Measurement.

[6] Mobili A., Belli A., Giosuè C., Pierpaoli M., Bastianelli L., Mazzoli A., Ruello M.L., Bellezze T., Tittarelli F. (2021). Mechanical, durability, depolluting and electrical properties of multifunctional mortars prepared with commercial or waste carbon-based fillers. Constr. Build. Mater..

[7] Lambert T.N., Chavez C.A., Hernandez-Sanchez B., Lu P., Bell N.S., Ambrosini A., Friedman T., Boyle T.J., Wheeler D.R., Huber D.L. (2009). Synthesis and Characterization of Titania-Graphene Nanocomposites. J. Phys. Chem..

[8] Chintalapudi K., Pannem R.M.R. (2020). The effects of Graphene Oxide addition on hydration process, crystal shapes, and microstructural transformation of Ordinary Portland Cement. J. Build. Eng..

[9] Karachevtsev V.A., Kurnosov N.V. (2019). The temperature dependence of electron transport in a composite film of graphene oxide with single-wall carbon nanotubes: An analysis and comparison with a nanotube film. Low Temp. Phys..

[10] Sim H.J., Li Z., Xiao P., Lu H. (2022). The Influence of Lateral Size and Oxidation of Graphene Oxide on Its Chemical Reduction and Electrical Conductivity of Reduced Graphene Oxide. Molecules.

[11] Fan X., Peng W., Li Y., Li X., Wang S., Zhang G., Zhang F. (2008). Deoxygenation of Exfoliated Graphite Oxide under Alkaline Conditions: A Green Route to Graphene Preparation. Adv. Mater..

[12] Yan S., He P., Jia D., Yang Z., Duan X., Wang S., Zhou Y. (2015). In Situ fabrication and characterization of graphene/geopolymer composites. Ceram. Int..

[13] Qureshi T.S., Panesar D.K. (2019). Impact of graphene oxide and highly reduced graphene oxide on cement based composites. Constr. Build. Mater..

[14] Yaseen S.A., Yiseen G.A., Li Z. (2019). Elucidation of Calcite Structure of Calcium Carbonate Formation Based on Hydrated Cement Mixed with Graphene Oxide and Reduced Graphene Oxide. ACS Omega.

[15] Kong X., Wang R., Zhang T., Sun R., Fu Y. (2022). Effects of graphene oxygen content on durability and microstructure of cement mortar composites. Constr. Build. Mater..

[16] Gholampour A., Valizadeh Kiamahalleh M., Tran D.N.H., Ozbakkaloglu T., Losic D. (2017). From Graphene Oxide to Reduced Graphene Oxide: Impact on the Physiochemical and Mechanical Properties of Graphene–Cement Composites. ACS Appl. Mater. Interfaces.

[17] Yan S., He P., Jia D., Yang Z., Duan X., Wang S., Zhou Y. (2016). Effects of treatment temperature on the reduction of GO under alkaline solution during the preparation of graphene/geopolymer composites. Ceram. Int..

[18] Yan S., He P., Jia D., Duan X., Yang Z., Wang S., Zhou Y. (2016). In Situ Processing of Graphene/Leucite Nanocomposite Through Graphene Oxide/Geopolymer. J. Am. Ceram. Soc..

[19] Liu R., Guo W., Sun B., Pang J., Pei M., Zhou G. (2015). Composites of rutile TiO_2_ nanorods loaded on graphene oxide nanosheet with enhanced electrochemical performance. Electrochim. Acta.

[20] Alaie M.M., Jahangiri M., Rashidi A.M., Asl A.H., Izadi N. (2015). A novel selective H_2_S sensor using dodecylamine and ethylenediamine functionalized graphene oxide. J. Ind. Eng. Chem..

[21] Abujabal M., Abunahla H., Mohammad B., Alazzam A. (2022). Tunable Switching Behavior of GO-Based Memristors Using Thermal Reduction. Nanomaterials.

[22] Grilli F., Gohari P.H., Zou S. (2022). Characteristics of Graphene Oxide for Gene Transfection and Controlled Release in Breast Cancer Cells. Int. J. Mol. Sci..

[23] Sengupta I., Kumar S.S.S., Pal S.K., Chakraborty S. (2020). Characterization of structural transformation of graphene oxide to reduced graphene oxide during thermal annealing. J. Mater. Res..

[24] Jung H. (2022). Multiple dispensing and photo-thermal reduction of graphene oxide solution for line patterning. Chem. Phys. Lett..

[25] Klemeyer L., Park H., Huang J. (2021). Geometry-Dependent Thermal Reduction of Graphene Oxide Solid. ACS Mater. Lett..

[26] Ruttanapun C., Phrompet C., Tuichai W., Karaphun A., Daengsakul S., Sriwong C. (2021). Influence of free electron charge and free extra framework anions in calcium aluminate@ rGO (CA@ rGO) cement composites with enhanced dielectric and electrochemical properties. J. Taiwan Inst. Chem. Eng..

[27] Zhang J., Scherer G.W. (2011). Comparison of methods for arresting hydration of cement. Cem. Concr. Res..

[28] Tunstall L.E., Scherer G.W., Prud’Homme R.K. (2021). A new hypothesis for air loss in cement systems containing fly ash. Cem. Concr. Res..

[29] Lavagna L., Bartoli M., Musso S., Suarez-Riera D., Tagliaferro A., Pavese M. (2022). A First Assessment of Carbon Nanotubes Grown on Oil-Well Cement via Chemical Vapor Deposition. Nanomaterials.

[30] Li P., Liu J., Her S., Nezhad E.Z., Lim S., Bae S. (2021). Synthesis of Highly-Dispersed Graphene Oxide Nanoribbons–Functionalized Carbon Nanotubes–Graphene Oxide (GNFG) Complex and Its Application in Enhancing the Mechanical Properties of Cementitious Composites. Nanomaterials.

[31] Darsanasiri A.G.N.D., Matalkah F., Ramli S., Al-Jalode K., Balachandra A., Soroushian P. (2018). Ternary alkali aluminosilicate cement based on rice husk ash, slag and coal fly ash. J. Build. Eng..

[32] Collier N.C., Sharp J.H., Milestone N.B., Hill J., Godfrey I. (2008). The influence of water removal techniques on the composition and microstructure of hardened cement pastes. Cem. Concr. Res..

[33] (2007). Test Method for Strength of Cement Paste (ISO Method).

[34] Ding F., Ji H., Chen Y., Herklotz A., Dörr K., Mei Y., Rastelli A., Schmidt O.G. (2010). Stretchable Graphene: A Close Look at Fundamental Parameters through Biaxial Straining. Nano Lett..

[35] Marcano D.C., Kosynkin D.V., Berlin J.M., Sinitskii A., Sun Z., Slesarev A., Alemany L.B., Lu W., Tour J.M. (2010). Improved Synthesis of Graphene Oxide. ACS Nano.

[36] Torrisi L., Cutroneo M., Manno D., Serra A., Torrisi A., Silipigni L. (2022). Proton beam dosimetry based on the graphene oxide reduction and Raman spectroscopy. Vacuum.

[37] Akbari E., Akbari I., Ebrahimi M.R. (2019). sp2/sp3 bonding ratio dependence of the band-gap in graphene oxide. Eur. Phys. J. B.

[38] Sharma N., Tomar S., Shkir M., Choubey R.K., Singh A. (2021). Study of Optical and Electrical Properties of Graphene Oxide. Mater. Today Proc..

[39] Hu L., Wang A., Wang W. (2022). Structural Evolution of Graphene Oxide and Its Thermal Stability During High Temperature Sintering. J. Wuhan Univ. Technol. Sci. Ed..

[40] Sharma N., Sharma V., Jain Y., Kumari M., Gupta R., Sharma S.K., Sachdev K. (2017). Synthesis and Characterization of Graphene Oxide (GO) and Reduced Graphene Oxide (rGO) for Gas Sensing Application. Macromol. Symp..

[41] Baskakov S.A., Baskakova Y.V., Kabachkov E.N., Vasilets V.N., Michtchenko A., Shulga Y.M. (2021). Influence of treatment with hydrazine and subsequent annealing on the composition and thermophysical properties of polytetrafluoroethylene–graphene oxide composite aerogel. Appl. Phys. A.

[42] Compton O.C., Jain B., Dikin D.A., Abouimrane A., Amine K., Nguyen S.T. (2011). Chemically Active Reduced Graphene Oxide with Tunable C/O Ratios. ACS Nano.

[43] Dave K., Park K.H., Dhayal M. (2015). Two-step process for programmable removal of oxygen functionalities of graphene oxide: Functional, structural and electrical characteristics. RSC Adv..

[44] Li C., Lu Y., Yan J., Yu W., Zhao R., Du S., Niu K. (2021). Effect of long-term ageing on graphene oxide: Structure and thermal decomposition. R. Soc. Open Sci..

[45] Wei J., Jia Z., Wang Y., Jiang Y., Miao Z., Zhou Y., Zhang H. (2021). Enhanced thermoelectric performance of low carbon cement-based composites by reduced graphene oxide. Energy Build..

[46] Zhai S., Pang B., Liu G., Zhang Y., Xu K., She W., Zhang Y. (2021). Investigation on preparation and multifunctionality of reduced graphene oxide cement mortar. Constr. Build. Mater..

[47] Xing-Wen J., Xin Z., Jun-Meng L., Ping W. (2018). Conductivity and conducting stability of copper-coated carbon-fiber-reinforced cement-based composite. Mater. Res. Express.

[48] Zhou Y., Ma Y., Li X., Bian C., Xiong Z., Sun Y., Chen H., Shen L. (2020). Tunable rGO network in polymer coating for enhancing barrier property. Mater. Res. Bull..

[49] Zhang N., She W., Du F., Xu K. (2020). Experimental Study on Mechanical and Functional Properties of Reduced Graphene Oxide/Cement Composites. Materials.

